# Child Mortality Estimation: A Global Overview of Infant and Child Mortality Age Patterns in Light of New Empirical Data

**DOI:** 10.1371/journal.pmed.1001299

**Published:** 2012-08-28

**Authors:** Michel Guillot, Patrick Gerland, François Pelletier, Ameed Saabneh

**Affiliations:** 1 Population Studies Center, University of Pennsylvania, Philadelphia, Pennsylvania, United State of America; 2 United Nations Population Division, New York, New York, United States of America; Umeå Centre for Global Health Research, Umeå University, Sweden

## Abstract

**Background:**

The under-five mortality rate (the probability of dying between birth and age 5 y, also denoted in the literature as U5MR and _5_ *q*_0_) is a key indicator of child health, but it conceals important information about how this mortality is distributed by age. One important distinction is what amount of the under-five mortality occurs below age 1 y (_1_ *q*_0_) versus at age 1 y and above (_4_ *q*_1_). However, in many country settings, this distinction is often difficult to establish because of various types of data errors. As a result, it is common practice to resort to model age patterns to estimate _1_ *q*_0_ and _4_ *q*_1_ on the basis of an observed value of _5_ *q*_0_. The most commonly used model age patterns for this purpose are the Coale and Demeny and the United Nations systems. Since the development of these models, many additional sources of data for under-five mortality have become available, making possible a general evaluation of age patterns of infant and child mortality. In this paper, we do a systematic comparison of empirical values of _1_ *q*_0_ and _4_ *q*_1_ against model age patterns, and discuss whether observed deviations are due to data errors, or whether they reflect true epidemiological patterns not addressed in existing model life tables.

**Methods and Findings:**

We used vital registration data from the Human Mortality Database, sample survey data from the World Fertility Survey and Demographic and Health Surveys programs, and data from Demographic Surveillance Systems. For each of these data sources, we compared empirical combinations of _1_ *q*_0_ and _4_ *q*_1_ against combinations provided by Coale and Demeny and United Nations model age patterns. We found that, on the whole, empirical values fall relatively well within the range provided by these models, but we also found important exceptions. Sub-Saharan African countries have a tendency to exhibit high values of _4_ *q*_1_ relative to _1_ *q*_0_, a pattern that appears to arise for the most part from true epidemiological causes. While this pattern is well known in the case of western Africa, we observed that it is more widespread than commonly thought. We also found that the emergence of HIV/AIDS, while perhaps contributing to high relative values of _4_ *q*_1_, does not appear to have substantially modified preexisting patterns. We also identified a small number of countries scattered in different parts of the world that exhibit unusually low values of _4_ *q*_1_ relative to _1_ *q*_0_, a pattern that is not likely to arise merely from data errors. Finally, we illustrate that it is relatively common for populations to experience changes in age patterns of infant and child mortality as they experience a decline in mortality.

**Conclusions:**

Existing models do not appear to cover the entire range of epidemiological situations and trajectories. Therefore, model life tables should be used with caution for estimating _1_ *q*_0_ and _4_ *q*_1_ on the basis of _5_ *q*_0_. Moreover, this model-based estimation procedure assumes that the input value of _5_ *q*_0_ is correct, which may not always be warranted, especially in the case of survey data. A systematic evaluation of data errors in sample surveys and their impact on age patterns of _1_ *q*_0_ and _4_ *q*_1_ is urgently needed, along with the development of model age patterns of under-five mortality that would cover a wider range of epidemiological situations and trajectories.

*Please see later in the article for the Editors' Summary.*

## Abstract 2

Michel Guillot and colleagues did a systematic evaluation to assess what proportion of under-five mortality occurs below age one compared with at age one and above, to determine how much observed values deviate from so called “model age patterns” of under-five mortality

## Editors' Summary

**Background:**

In 2000, world leaders agreed on eight Millennium Development Goals designed to end extreme poverty by 2015. The fourth of these goals—MDG 4—aims to reduce under-five mortality (the number of children who die before their fifth birthday) to a third of its 1990 level by 2015. A key indicator used to monitor progress towards this target is the under-five mortality rate (the probability of a child dying before his/her fifth birthday, also denoted as U5MR or _5_ *q*_0_). In developed countries, data collected through vital registration systems (which record all births and deaths) are used to calculate _5_ *q*_0_. However, developing countries, which are where most under-five deaths occur, rarely have vital registration systems, and _5_ *q*_0_ is estimated using data collected by programs such as the World Fertility Survey (WFS) and Demographic and Health Surveys (DHS), which conduct nationally representative surveys that ask a sample of women about their living and dead children.

**Why Was This Study Done?:**

Although _5_ *q*_0_ is a key indicator of child health, it conceals important information about the age distribution of child deaths. Public health experts need to know the distribution of _5_ *q*_0_ with respect to _1_ *q*_0_ (the probability that an infant will die before age one) and _4_ *q*_1_ (the probability that a child reaching age one will die below age five) to help them reduce child mortality. At a given level of _5_ *q*_0_, high values of _1_ *q*_0_ indicate high levels of death from congenital (inherited) anomalies and conditions that occur around the time of birth; these deaths can be reduced by improving the care of women during pregnancy and childbirth and the care of newborn babies. By contrast, at a given level of _5_ *q*_0_, high values of _4_ *q*_1_ indicate high levels of death from infectious diseases; these deaths can be reduced by, for example, introducing immunization programs. _1_ *q*_0_ and _4_ *q*_1_ are usually estimated from observed (empirical) values of _5_ *q*_0_ using the Coale and Demeny or United Nations (UN) “model life tables” (mathematical models of the variation of mortality with age), which were constructed in 1966 and 1982, respectively, using the best data available. Since their construction, additional sources of data about under-five mortality have become available; in this study, the researchers systematically compare global empirical values of _1_ *q*_0_ and _4_ *q*_1_ with values obtained using model life tables.

**What Did the Researchers Do and Find?:**

The researchers compared empirical combinations of _1_ *q*_0_ and _4_ *q*_1_ (estimated using vital registration data, WFS and DHS data, and data from Demographic Surveillance Sites in sub-Saharan Africa) with the combinations derived from _5_ *q*_0_ using the Coale and Demeny and UN model life tables. The empirical values mainly fell within the range provided by these tables, but there were important exceptions. For example, empirical values of _4_ *q*_1_ relative to _1_ *q*_0_ tended to be above the range provided by the model life tables for sub-Saharan African countries. This pattern was mainly because of epidemiological reasons (epidemiology is the study of disease patterns in populations), such as the occurrence of diseases such as malaria, measles, and diarrhea that generate excess mortality among children older than one year. Interestingly, the emergence of HIV does not seem to have substantially modified preexisting patterns of _1_ *q*_0_ versus _4_ *q*_1_. Importantly, the researchers also show that populations often experience changes in the age patterns of infant and child mortality as they experience an overall decline in mortality.

**What Do These Findings Mean?:**

These findings suggest that the existing model life tables do not cover the entire global range of epidemiological situations and trajectories and must, therefore, be used with caution for estimating _1_ *q*_0_ and _4_ *q*_1_ on the basis of _5_ *q*_0_. The development of new model age patterns of under-five mortality that cover a wider range of epidemiological situations should improve this situation, but a systematic analysis of data errors in sample surveys and the impact of such errors on estimates of _1_ *q*_0_ and _4_ *q*_1_ is also urgently needed to ensure that public health experts have access to accurate information on child mortality. Importantly, this overview shows that a wide range of _1_ *q*_0_ and _4_ *q*_1_ combinations can occur at a given level of _5_ *q*_0_. Because the level of _4_ *q*_1_ relative to _1_ *q*_0_ provides important information about the disease processes occurring in a population, this finding highlights the importance of determining _1_ *q*_0_ and _4_ *q*_1_ as well as _5_ *q*_0_ whenever possible.

**Additional Information:**

Please access these websites via the online version of this summary at http://dx.doi.org/10.1371/journal.pmed.1001299.

- This paper is part of a collection of papers on Child Mortality Estimation Methods published in *PLOS Medicine*
- The United Nations Childrens Fund (UNICEF) works for children's rights, survival, development, and protection around the world; it provides information on Millennium Development Goal 4, and its Childinfo website provides detailed statistics about child survival and health, including a description of the United Nations Inter-agency Group for Child Mortality Estimation; the 2011 UN IGME report Levels & Trends in Child Mortality is available
- The World Health Organization also has information about Millennium Development Goal 4 and provides estimates of child mortality rates (some information in several languages)
- Further information about the Millennium Development Goals is available
- Information is also available about the Human Mortality Database, which holds vital registration data; the World Fertility Survey program; the Demographic and Health Surveys program; and model life tables

## Introduction

The under-five mortality rate (the probability of dying between birth and age 5 y, also denoted in the literature as U5MR and _5_ *q*_0_) is a key mortality indicator that is routinely used to track progress in the area of child health and is featured prominently among the Millennium Development Goals [1],[2]. This indicator, however, conceals important information about how this mortality is distributed by age. One important distinction is what portion of the under-five mortality occurs below age 1 y (the infant mortality rate [_1_ *q*_0_]) versus at age 1 y and above (the child mortality rate [_4_ *q*_1_]).

The distribution of _5_ *q*_0_ with respect to _1_ *q*_0_ (the probability that a newborn will die below age one) and _4_ *q*_1_ (the probability that a child reaching age one will die below age 5) is important, because it reveals useful information about a population's epidemiological context [3],[4]. At a given level of _5_ *q*_0_, high values of _1_ *q*_0_ reflect high levels of mortality from congenital anomalies and perinatal conditions, since these causes are responsible for most mortality during the early days of life. These conditions are most affected by factors such as obstetric practice and neonatal care [5]–[8]. By contrast, infectious diseases have an “older” age pattern within the under-five age range, because during the first few months of life a newborn is protected to some extent by the passive immunity inherited from the mother [9]–[12]. Therefore, at a given level of _5_ *q*_0_, high values of _4_ *q*_1_ reflect high levels of mortality from infectious diseases. These diseases are affected by a wide range of factors such as environmental characteristics, sanitation, diet, immunization, child care practices, and health care.

In spite of its epidemiological significance, the age pattern of _1_ *q*_0_ and _4_ *q*_1_ is difficult to establish in countries that lack reliable vital registration systems. In such countries, _1_ *q*_0_, _4_ *q*_1_, and _5_ *q*_0_ are derived primarily from birth histories collected as part of sample surveys such as in the Demographic and Health Surveys (DHS) program. These birth histories, however, are subject to a number of errors, including omission of deaths and age misreporting errors. Even though _5_ *q*_0_ is not free of errors (e.g., it is affected by omission of under-five deaths), it is generally considered a more reliable indicator than _1_ *q*_0_ and _4_ *q*_1_ in sample surveys, because unlike _1_ *q*_0_ and _4_ *q*_1_, it is not subject to incorrect reporting of whether a death occurred before or after the child's first birthday. Another reason is that the larger amount of mortality exposure embodied in _5_ *q*_0_ attenuates the amount of random sampling error for that indicator.

Given the advantages of _5_ *q*_0_, a common approach to the estimation of _1_ *q*_0_ and _4_ *q*_1_ consists of using model age patterns of mortality such as those embodied in model life tables. Model life tables represent regularities in relationships among mortality indicators at different ages observed in populations with high data quality. Taking advantage of these regularities, one can derive a missing or flawed mortality indicator on the basis of another mortality indicator believed to be less subject to errors. This approach is often used to estimate _1_ *q*_0_ and _4_ *q*_1_ on the basis of _5_ *q*_0_. It relies on two premises: (1) the observed value of _5_ *q*_0_ used as input is correct; (2) the chosen model adequately represents the relationship between _1_ *q*_0_ and _4_ *q*_1_ in the population of interest.

The model life tables that are most often used for this purpose are the Coale and Demeny [13],[14] and the United Nations (UN) model life tables [15]. These models are based on the best data that were available at the time they were constructed (1966 for Coale and Demeny, and 1982 for the UN). Since that time, however, the amount of available data on infant and child mortality has greatly expanded. A wealth of information is now available for less developed countries. Also, levels of infant and child mortality have declined to unprecedented levels in several countries of the world. These developments make it possible to conduct an overview of empirical age patterns of infant and child mortality worldwide, and assess the relevance of existing models.

In this paper, we do a systematic comparison of empirical values of _1_ *q*_0_ and _4_ *q*_1_ against Coale and Demeny and UN model age patterns. We examine patterns of deviation from these models and discuss these deviations in terms of two main reasons for it. The first reason is data errors. Indeed, some of the data points that we present in this study may be affected by errors such as omission of infant deaths, among which neonatal deaths seem to be particularly at risk, and misreporting of ages at death, especially age heaping around the first birthday. These sources of error seem particularly relevant in reference to the DHS data [16]; they make the observed value of _4_ *q*_1_ against _1_ *q*_0_ appear higher than what would be predicted by the population's true _1_ *q*_0_ versus _4_ *q*_1_ relationship. The second reason is epidemiological conditions. The newly available data may in fact be correct and represent true epidemiological patterns not addressed in existing model life tables.

This paper builds on previous work by Garenne [9], Blacker et al. [17], United Nations [18]; Sullivan et al. [19], Hill [16], Bicego and Ahmad [20], and Jasseh [21]. We expand this existing literature by taking a global approach, examining the experience of countries in both the more and less developed regions, and by examining both historical and current patterns. We also provide new information about the possible contribution of poor data quality to observed deviations. Among other epidemiological explanations, we discuss the possible contribution of HIV/AIDS to the observed mortality patterns.

## Methods

### Data Sources

The data used in this paper come from two main sources. The first source is the Human Mortality Database (HMD), which consists of high-quality life tables mostly from Western countries. The earliest year for which data are available is 1751 (for Sweden), and the most recent year for each country is 2009 or 2010. This database represents high-quality vital registration. In this analysis, we used 838 quinquennial life tables covering 39 countries or areas. (For the purpose of this analysis we included whole populations only [i.e., we excluded “civilian population” for France and England and Wales, and “Maori” and “non-Maori” for New Zealand], and we used the mutually exclusive geographic areas providing the longest time series [i.e., German Democratic Republic and Federal Republic of Germany for 1956–2008 instead of Germany since 1990, England and Wales for 1841–2009, Northern Ireland since 1922, and Scotland since 1855 instead of United Kingdom since 1922]. Taiwan was excluded because of the poor quality of its infant mortality information [22].)

In countries lacking reliable vital registration data, large-scale nationally representative cross-sectional household sample surveys have been conducted since the mid-1970s. They provide complete maternal or birth histories, allowing the direct computation of mortality rates through deaths and exposure up to 25 y prior to the survey date. Several international survey programs have contributed to this endeavor [23]. They rely on high-quality survey instruments and comparable methodology, and provide widely used infant and child mortality data. In this study we focused our analysis on two of these major survey data sources: (1) 27 World Fertility Survey (WFS) surveys conducted between 1974 and 1981 in developing regions (and spanning the retrospective period 1950–1981), and (2) 217 nationally representative DHS surveys conducted between 1985 and 2010 (and covering the retrospective period 1963–2010). In addition to the standard DHS surveys, all interim DHS surveys and surveys by the AIDS Indicator Survey and the Malaria Indicator Survey that collected full retrospective birth histories were also included. The 1986 Nigeria DHS survey conducted only in Ondo State was excluded. The 2008–2009 Albania DHS survey was not included in this analysis because the region is already well represented by the HMD. The _1_ *q*_0_ and _4_ *q*_1_ values that we give in this paper are based on deaths and exposure of children within these particular age ranges. Following standard DHS practice, we present estimates by 5-y periods for 15 y prior to the survey (e.g., 0–4 y, 5–9 y, and 10–14 y before the survey) [24],[25].

Altogether, the WFS and DHS data provided us with 689 unique pairs of observations (country×period), covering 86 countries in developing regions (15 in Asia/Oceania, 16 in Central and Latin America, eight in the Middle East and Northern Africa, and 39 in sub-Saharan Africa) or from the former Soviet Union (8).

While the bulk of data comes from the HMD and sample surveys, we also present some information from Demographic Surveillance System (DSS) sites located in sub-Saharan African countries. These data are mainly from small rural communities and therefore are not representative at the national level like DHS data. However, they do provide comprehensive samples of good-quality demographic rates as they monitor systematically and with great care all vital events and populations in the study areas through annual (or even more frequent) visits [26]. Altogether, our DSS data analysis reviews 127 mortality rates for different periods from 1930 to 2006 across 28 sites in sub-Saharan Africa (18 in western Africa, nine in eastern Africa, and one in southern Africa). The complete list of sites is provided in Text S1, together with detailed information about the other data sources used in this paper.

In this paper, we present mortality indicators for both sexes combined, rather than by sex. This approach is motivated by the fact that discussions of global trends in infant and child mortality typically focus on information for both sexes combined. Also, approaches using model life tables in the area of infant and child mortality estimation typically rely on mortality information for both sexes combined. Moreover, this merging of males and females allows us to increase the robustness of our analysis and minimize issues with rates computed for smaller sample sizes from surveys or from vital registration for smaller populations (especially at low mortality levels).

### Model Life Tables

The two sets of model life tables that we focus on in this paper are the Coale and Demeny and the UN systems. This choice is motivated by the fact that these two sets are the most commonly used model age patterns and are integrated in mortality estimation packages such as MortPak [27]. Coale and Demeny and the UN model life tables summarize variations in age-specific mortality using two dimensions. The first dimension is “family” or “pattern” and represents life tables with similar age patterns of mortality, based on geographical clusters of countries used as the model's empirical basis. The second dimension is “level” and represents how age-specific mortality rates vary within a family as the overall level of mortality changes. In this paper, we present results for the following families: “North,” “South,” “East,” and “West” families for the Coale and Demeny model, and “Latin American,” “Chilean,” and “General” patterns for the UN model. (For ease of presentation, the UN “South Asian” and “Far Eastern” patterns are not included in the figures as they do not add much additional information relative to other patterns.) We present model life table values for all available mortality levels, and also include values for low mortality levels that were not included in the original set. The low mortality values are derived from the original set using standard models for extrapolating mortality [28],[29]. Model life tables for both sexes combined are obtained from sex-specific model life tables by calculating a weighted average of male and female values of _4_ *q*_1_ predicted at a given level of _1_ *q*_0_ while assuming a sex ratio at birth of 1.05.

In this paper, we focus on the relationship between _4_ *q*_1_ and _1_ *q*_0_, instead of the relationship between _5_ *q*_0_ and _1_ *q*_0_. The relationship between _5_ *q*_0_ and _1_ *q*_0_ is a byproduct of the relationship between the _4_ *q*_1_ and _1_ *q*_0_. Indeed, the three indicators are related with one another through the equation _5_ *q*_0_ = 1−(1−_1_ *q*_0_)(1−_4_ *q*_1_). We decided to focus on the relationship between _4_ *q*_1_ and _1_ *q*_0_, because these two indicators have no age overlap, which generates more variance and makes patterns of deviation easier to detect.

Given the large number of country-years analyzed in this paper, and the sampling error attached to some of the estimates, it is natural to find some amount of scatter in the relationship between _1_ *q*_0_ and _4_ *q*_1_. Rather than focusing on individual outliers, we examine here broad patterns of deviation by country/region and period, and whether these patterns can be validated with other independent data sources.

## Results

### HMD Data

**Figure 1:**
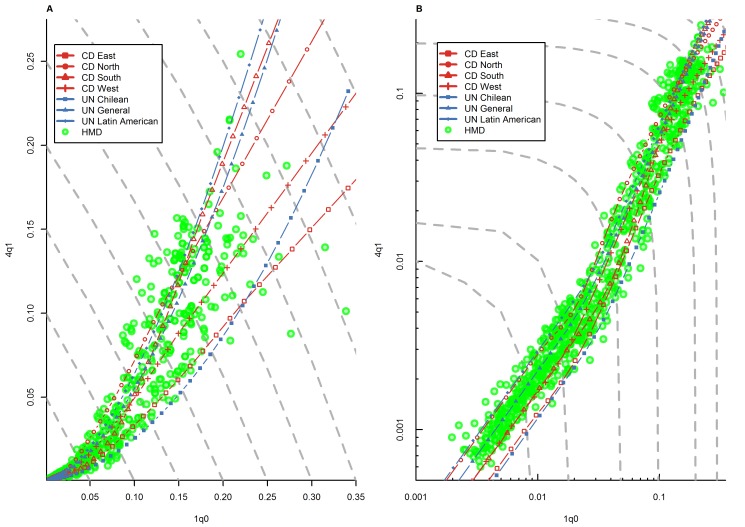
Relationship between _1_ *q*_0_ and _4_ *q*_1_ in country-years of the HMD. (A) Linear scale; (B) logarithmic scale. Gray dashed lines represent isoclines for combinations of _1_ *q*_0_ and _4_ *q*_1_ that produce an identical level of _5_ *q*_0_. CD, Coale and Demeny.

Figure 1 presents the _1_ *q*_0_ versus _4_ *q*_1_ relationship for both sexes combined in all countries included the HMD, together with the _1_ *q*_0_ versus _4_ *q*_1_ relationship modeled in the Coale and Demeny and UN systems of model life tables. Figure 1 and subsequent figures present isoclines for combinations of _1_ *q*_0_ and _4_ *q*_1_ that produce an identical level of _5_ *q*_0_. These _5_ *q*_0_ isoclines allow one to visualize the amount of variation in _1_ *q*_0_ and _4_ *q*_1_ that can arise at a given level of _5_ *q*_0_. Prediction errors for observed values of _4_ *q*_1_ that fall outside the range predicted by model life tables are summarized in Table 1.

**Table 1:** Root mean square error for differences between observed values of _4_ *q*_1_ and values of _4_ *q*_1_ predicted using model life tables, with _1_ *q*_0_ as an input.

Region (Data Source)	*n*	Percent Points outside Range	Percent Points below Range	Percent Points above Range	Root Mean Square Error outside Range	Root Mean Square Error below Range	Root Mean Square Error above Range
Europe/North America/Japan (HMD)	838	16.7%	1.6%	15.2%	0.01247	0.00335	0.01311
Sub-Saharan Africa (DHS/WFS)	315	66.0%	0.0%	66.0%	0.03332	n/a	0.03332
Asia (DHS/WFS)	114	27.2%	2.6%	24.6%	0.00637	0.00453	0.00653
Former Soviet Union (DHS/WFS)	30	33.3%	30.0%	3.3%	0.00186	0.00194	0.00092
Latin America and the Caribbean (DHS/WFS)	132	15.9%	9.1%	6.8%	0.00490	0.00414	0.00577
Middle East and northern Africa (DHS/WFS)	98	15.3%	5.1%	10.2%	0.01418	0.00063	0.01736

Only prediction errors above the maximum model-predicted value or below the minimum model-predicted value are included.

n/a, not applicable.

Figure 1 shows that, overall, country-years represented in the HMD fit remarkably well within the range modeled by these two model life table systems. This is not very surprising, because many of the country-years presented on this graph were actually included in Coale and Demeny's empirical basis. Data for some historical periods that include high levels of _1_ *q*_0_ not represented in Coale and Demeny's data also appear to fit well within the range predicted by the two models. However, we do note that for _1_ *q*_0_ levels between 100 and 200 deaths per 1,000 births, a number of country-years appear to have _4_ *q*_1_ levels above the range predicted by model life tables. (These data points include mostly Belgium, England and Wales, Scotland, and Norway during the second half of the 19th century.) Data points for low mortality levels, many of which were included neither in the UN nor the Coale and Demeny systems, also appear to fit well within the range, although at these levels the cloud of points tends to cluster around the higher side of the model life table range.

**Figure 2:**
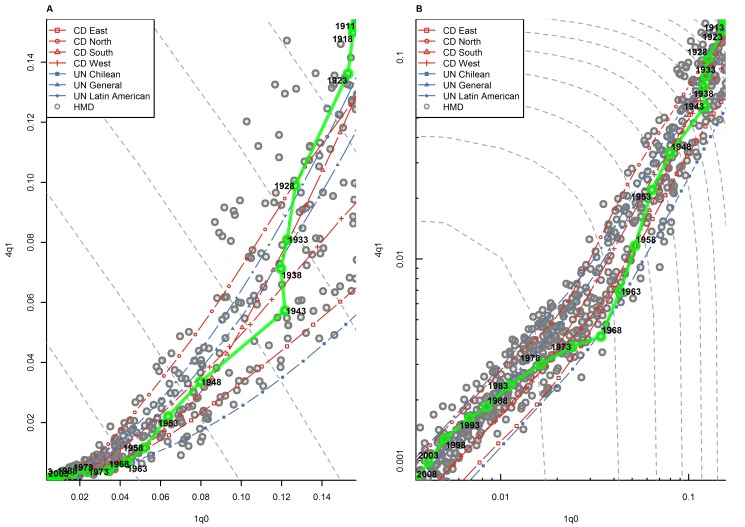
Relationship between _1_ *q*_0_ and _4_ *q*_1_ in Spain (1908–2009). (A) Linear scale; (B) logarithmic scale. The gray dots represent other country-years in the HMD. Gray dashed lines represent isoclines for combinations of _1_ *q*_0_ and _4_ *q*_1_ that produce an identical level of _5_ *q*_0_. CD, Coale and Demeny.

An important feature of this analysis is that many countries in the HMD appear to change families as they experience declines in _1_ *q*_0_ mortality levels. A typical example of this pattern is Spain, presented in Figure 2. At high levels of infant mortality, _4_ *q*_1_ in Spain appears on the higher side of the model life table range, at or above the Coale and Demeny North or the UN Chilean pattern. As infant mortality declines, however, _4_ *q*_1_ declines faster than what model patterns would predict, including even the South model, which offers the fastest trajectory of relative _4_ *q*_1_ decline. As a result, _4_ *q*_1_ values progressively move towards the lower part of the model life table range. As _1_ *q*_0_ reaches a value of about 35 deaths per 1,000 births around the year 1968, an abrupt change of slope occurs, and _4_ *q*_1_ starts declining more slowly, relative to _1_ *q*_0_, than what model patterns would predict. This trajectory is typical of countries with long time series, such as Sweden, France, Belgium, and Italy. Indeed, many countries experienced an abrupt change of slope around 1970. This pattern may be interpreted as arising from (1) improved obstetric practices and neonatal care [6], which may accelerate the decline of _1_ *q*_0_, and (2) a slowdown in the decline of _4_ *q*_1_ as this indicator reaches threshold values.

This example illustrates the difficulty of modeling age patterns of mortality, because these patterns result from many influences that can change over time in ways that are difficult to predict. To some extent, model life tables also exhibit a change of slope around _1_ *q*_0_ values of 30 deaths per 1,000 births, as shown in Figure 1B. But for many countries in the HMD, at high levels of mortality, declines in _4_ *q*_1_ relative to _1_ *q*_0_ have been faster than predicted by model patterns. At low levels of mortality, declines in _4_ *q*_1_ relative to _1_ *q*_0_ have been slower than predicted. As a result, many countries appear to change families as they experience mortality decline. Families of model life tables are meant to describe groups of countries that retain distinct features of the age pattern of mortality at various levels of mortality. In reality, however, a country's distinct features are not set in stone and can change rapidly overtime. The implications for users of model life tables is that one should be cautious about applying the same family of model tables over long periods of time, because changes in mortality conditions can affect the trajectory of _4_ *q*_1_ relative to _1_ *q*_0_ in ways that are not well addressed by these models.

### WFS and DHS Data

**Figure 3:**
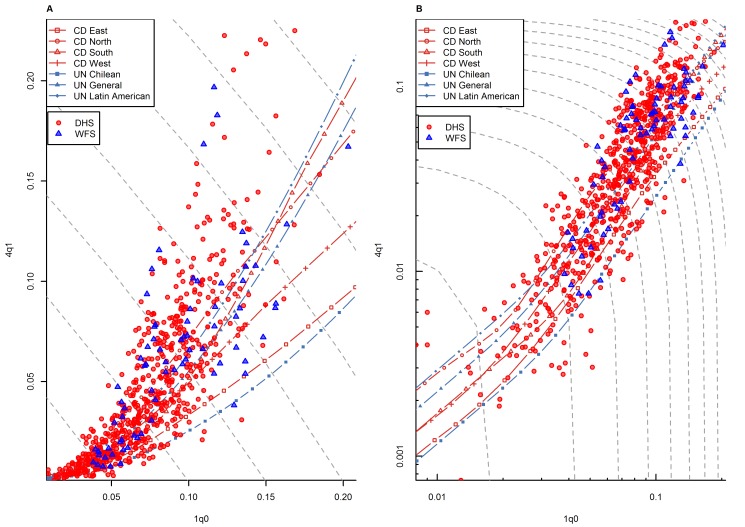
Relationship between _1_ *q*_0_ and _4_ *q*_1_ in country-years of the WFS and DHS surveys: all regions. (A) Linear scale; (B) logarithmic scale. Gray dashed lines represent isoclines for combinations of _1_ *q*_0_ and _4_ *q*_1_ that produce an identical level of _5_ *q*_0_. CD, Coale and Demeny.

Figure 3 shows the _1_ *q*_0_ versus _4_ *q*_1_ relationship in all country-years for which WFS- or DHS-based estimates are available. Unlike data points from the HMD, these points represent the experience of countries in less developed regions. They need to be interpreted with more caution, because, as mentioned above, they are based on retrospective data subject to response bias, while the HMD data are based on vital registration systems that meet HMD's high quality standards. WFS and DHS data are also based on samples rather than exhaustive registration systems, and are therefore likely to be more scattered than in the case of the HMD. With these caveats in mind, we see that while many country-years appear to fall within the range predicted by model life tables, data points are clearly not as well aligned with model life tables as in the case of the HMD. In particular, data points at higher levels of _1_ *q*_0_ tend to be located above range.

**Figure 4:**
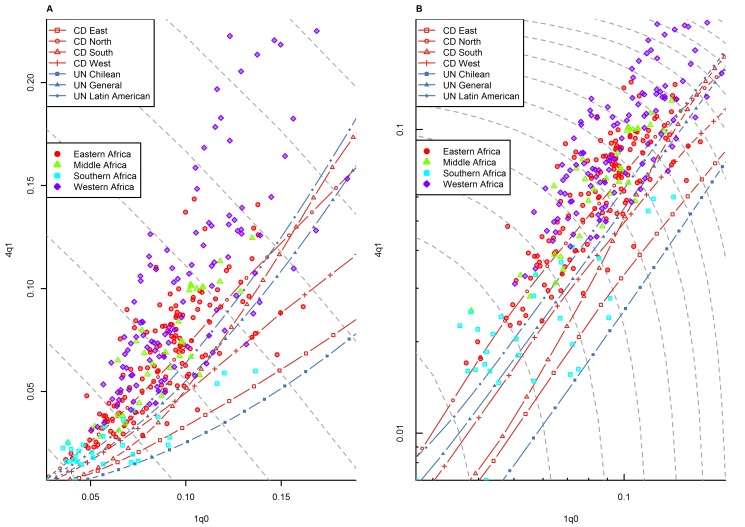
Relationship between _1_ *q*_0_ and _4_ *q*_1_ in country-years of the WFS and DHS surveys: sub-Saharan Africa. (A) Linear scale; (B) logarithmic scale. Gray dashed lines represent isoclines for combinations of _1_ *q*_0_ and _4_ *q*_1_ that produce an identical level of _5_ *q*_0_. CD, Coale and Demeny.

Figures 4–8 present the same information by broad regions. There is a clear regional pattern in observed deviations from model life tables. Sub-Saharan Africa (Figure 4) clearly stands out in its tendency to present above-range values of _4_ *q*_1_. As shown in Table 1, this region has the highest percentage of points falling above range (66.0%), and it has no point falling below range. This pattern is present in all sub-regions of sub-Saharan Africa for which WFS/DHS data are available, except the southern Africa region (which includes Botswana, Lesotho, Namibia, South Africa, and Swaziland). We discuss this pattern in more detail in the next section.

**Figure 5 pmed-1001299-g005:**
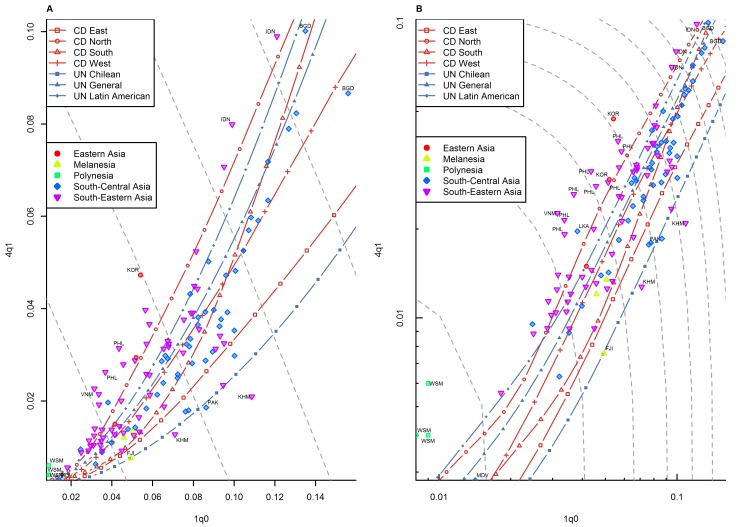
Relationship between _1_ *q*_0_ and _4_ *q*_1_ in country-years of the WFS and DHS surveys: Asia. (A) Linear scale; (B) logarithmic scale. Observations outside the range of values from the model life tables are labeled with their International Organization for Standardization (ISO) country codes as follows: BGD, Bangladesh; FJI, Fiji; IDN, Indonesia; KHM, Cambodia; KOR, Republic of Korea; LKA, Sri Lanka; MDV, Maldives; PAK, Pakistan; PHL, Philippines; VNM, Viet Nam; WSM, Samoa. Gray dashed lines represent isoclines for combinations of _1_ *q*_0_ and _4_ *q*_1_ that produce an identical level of _5_ *q*_0_. CD, Coale and Demeny.

**Figure 6 pmed-1001299-g006:**
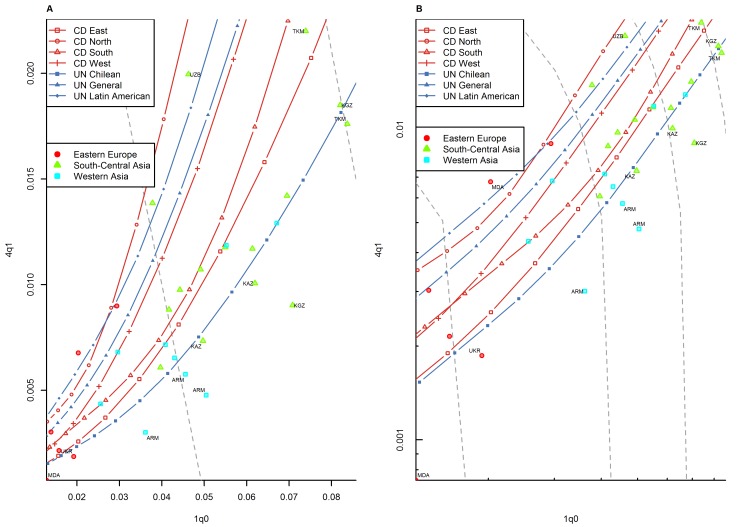
Relationship between _1_ *q*_0_ and _4_ *q*_1_ in country-years of the WFS and DHS surveys: former Soviet Union. (A) Linear scale; (B) logarithmic scale. Observations outside the range of values from the model life tables are labeled with their ISO country codes as follows: ARM, Armenia; KAZ, Kazakhstan; KGZ, Kyrgyzstan; MDA, Republic of Moldova; TKM, Turkmenistan; UKR, Ukraine; UZB, Uzbekistan. Gray dashed lines represent isoclines for combinations of _1_ *q*_0_ and _4_ *q*_1_ that produce an identical level of _5_ *q*_0_. CD, Coale and Demeny.

**Figure 7 pmed-1001299-g007:**
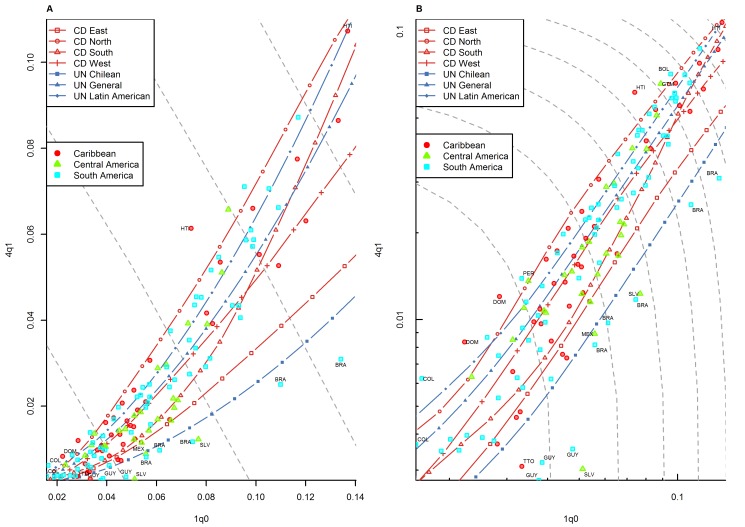
Relationship between _1_ *q*_0_ and _4_ *q*_1_ in country-years of the WFS and DHS surveys: Latin America and the Caribbean. (A) Linear scale; (B) logarithmic scale. Observations outside the range of values from the model life tables are labeled with their ISO country codes as follows: BOL, Bolivia; BRA, Brazil; COL, Colombia; DOM, Dominican Republic; GTM, Guatemala; GUY, Guyana; HTI, Haiti; MEX, Mexico; PER, Peru; SLV, El Salvador; TTO, Trinidad and Tobago. Gray dashed lines represent isoclines for combinations of _1_ *q*_0_ and _4_ *q*_1_ that produce an identical level of _5_ *q*_0_. CD, Coale and Demeny.

**Figure 8:**
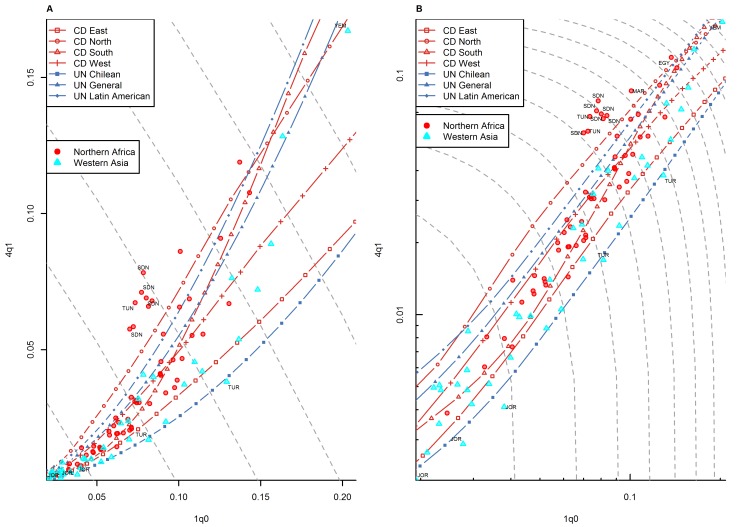
Relationship between _1_ *q*_0_ and _4_ *q*_1_ in country-years of the WFS and DHS surveys: Middle East and northern Africa. (A) Linear scale; (B) logarithmic scale. Observations outside the range of values from the model life tables are labeled with their ISO country codes as follows: EGY, Egypt; JOR, Jordan; MAR, Morocco; SDN, Sudan; TUN, Tunisia; TUR, Turkey; YEM, Yemen. Gray dashed lines represent isoclines for combinations of _1_ *q*_0_ and _4_ *q*_1_ that produce an identical level of _5_ *q*_0_. CD, Coale and Demeny.

For other regions of the world, the WFS/DHS data are better aligned with existing model patterns. In Asia (Figure 5), the large majority of country-years fall within range, with a few exceptions, including the Philippines and Viet Nam. Figure 6 shows countries of the former Soviet Union where DHS surveys have been conducted, which include mostly republics of the Caucasus and central Asia. These countries have an unusual tendency to fall below the range predicted by existing model life tables. Indeed, Table 1 shows that this region has the highest percentage (30.0%) of points falling below range. Latin American and Caribbean countries (Figure 7) are clearly within range, although we do note that a few country-years, most notably for Brazil, fall towards the lower end of the range. Countries of the Middle East and northern Africa (Figure 8) also fall mostly within range. (One clear exception is Sudan, which exhibits a pattern similar to that of many countries of sub-Saharan Africa.) We also note that Turkey and Jordan have consistent tendencies to experience relatively low values of _4_ *q*_1_, at or below those predicted by the Chilean model.

**Figure 9:**
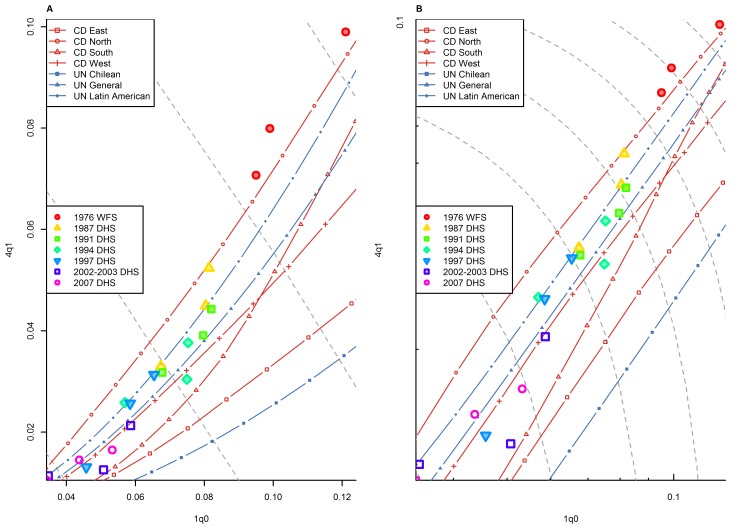
Relationship between _1_ *q*_0_ and _4_ *q*_1_ in Indonesia (WFS and DHS data). (A) Linear scale; (B) logarithmic scale. Gray dashed lines represent isoclines for combinations of _1_ *q*_0_ and _4_ *q*_1_ that produce an identical level of _5_ *q*_0_. CD, Coale and Demeny.

As in the case of countries in the HMD, many countries that fall within the range predicted by model life tables change families as they experience declines in mortality. This is more difficult to establish than in the case of countries covered by the HMD, because periods of observation are shorter. Also, time series come from surveys with different sample sizes, and likely, different levels of data quality. Nonetheless, we present in Figure 9 the example of Indonesia, which appears to have experienced declines in _4_ *q*_1_ relative to _1_ *q*_0_ that are faster than predicted by model age patterns, with a progressive shift from the North towards the West pattern. Such a change from relatively high to relatively low values of _4_ *q*_1_ as _1_ *q*_0_ declines is rather common in WFS/DHS countries, and in some ways it parallels the experience of Spain at similar levels of mortality. Like in the case of Spain, this example illustrates the inadequacy of model life tables for representing individual trajectories of _4_ *q*_1_ versus _1_ *q*_0_. (One should note that this fast decline in _4_ *q*_1_ relative to _1_ *q*_0_ is reflected to some extent in Coale and Demeny's South pattern, though in the example of Indonesia the recorded levels of _4_ *q*_1_ are higher.)

### Understanding the Pattern in Sub-Saharan Africa

As said earlier, the most striking deviations from model life table patterns are found in sub-Saharan Africa. Indeed, Table 1 shows that this region has by the far the highest amount of prediction error, all which occurs above range. Figure 4 shows that this pattern is not limited to western African countries, but is also present in eastern and middle Africa. This confirms and updates findings by Jasseh [21]. We note that the existence of this pattern is well known in the case of western Africa [9],[10],[30]–[32], but that it is somewhat underappreciated in the case of other regions of sub-Saharan Africa.

**Figure 10:**
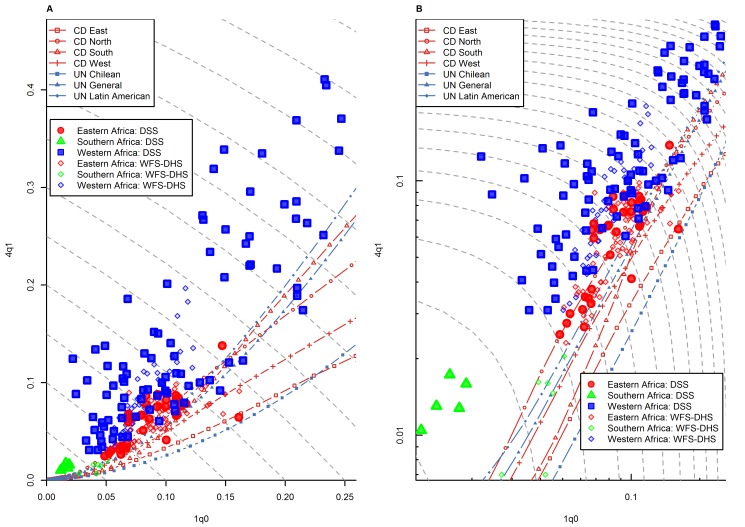
Relationship between _1_ *q*_0_ and _4_ *q*_1_ in DSS data versus WFS/DHS data for sub-Saharan African countries where DSS sites are located. (A) Linear scale; (B) logarithmic scale. Gray dashed lines represent isoclines for combinations of _1_ *q*_0_ and _4_ *q*_1_ that produce an identical level of _5_ *q*_0_. CD, Coale and Demeny.

Data quality problems seem an unlikely explanation for this pattern, because unusually high levels of _4_ *q*_1_ are also found in DSS data from the same region [21]. As discussed earlier, although these data are not representative of entire countries, they are considered to be reliable, because they result from intensive, prospective data collection schemes. Figure 10 presents the _1_ *q*_0_ versus _4_ *q*_1_ relationship from a number of DSS sites throughout sub-Saharan Africa. (For comparison, we also show national-level WFS/DHS information for the countries where these DSS sites are located.) The geographical representation is not the same as in Figure 4, but nonetheless we see a widespread pattern of above-range values of _4_ *q*_1_ in western Africa but also for some DSS sites in eastern Africa. The only DSS site in southern Africa included in this analysis is Agincourt (South Africa), and it also presents above-range values of _4_ *q*_1_.

**Figure 11:**
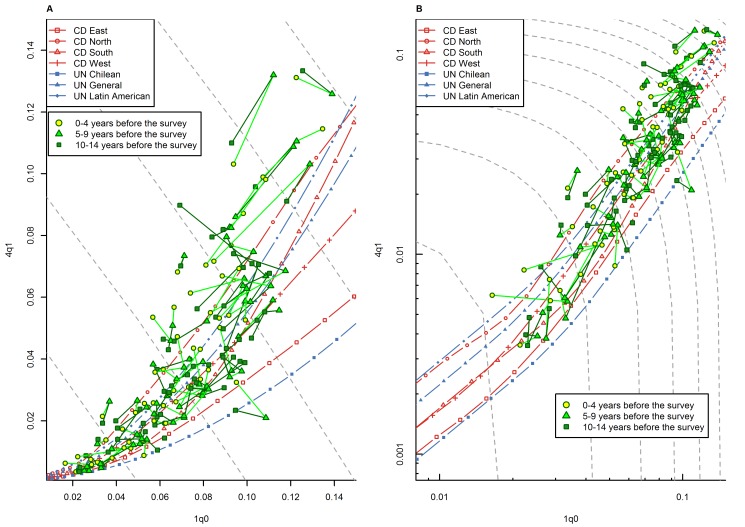
Relationship between _1_ *q*_0_ and _4_ *q*_1_ in WFS/DHS data from sub-Saharan African countries, by duration of the retrospective period. (A) Linear scale; (B) logarithmic scale. These data include only quinquennial calendar periods for which three pairs of _1_ *q*_0_ versus _4_ *q*_1_ country-specific estimates from different surveys were available, where the retrospective period was 0–4, 5–9, or 10–14 y prior to the survey. Points referring to the same country and calendar period are linked with one another by solid lines. Gray dashed lines represent isoclines for combinations of _1_ *q*_0_ and _4_ *q*_1_ that produce an identical level of _5_ *q*_0_. CD, Coale and Demeny.

The existence of a widespread pattern of relatively high _4_ *q*_1_ values in sub-Saharan Africa is further supported by an analysis of the WFS/DHS data in which we made the distinction between mortality information derived from recent retrospective periods, and information derived from more distant retrospective periods. Recall bias, including omission of births and deaths, and age heaping, are likely to be more severe for events that occurred long ago than for more recent events [33],[34]. Thus, if response bias is contributing to the observed high values of _4_ *q*_1_ relative to _1_ *q*_0_, we should expect above-range deviations to be larger for estimates derived from retrospective reports that are more distant in time, compared to estimates applying to the same calendar period but derived from more recent retrospective reports. Figure 11 presents WFS/DHS estimates of _1_ *q*_0_ versus _4_ *q*_1_ for quinquennial calendar periods for which three pairs of _1_ *q*_0_ versus _4_ *q*_1_ country-specific estimates were available: estimates based on reports for 0–4, 5–9, and 10–14 y prior to the survey. These three pairs, which are linked in Figure 11, were derived from three different surveys for which there was calendar period overlap between different retrospective reports. Since the three pairs referred to the same calendar period, discrepancies between them can only arise from bias or random error. In particular, time trends in _1_ *q*_0_ versus _4_ *q*_1_ cannot explain these discrepancies. Figure 11 shows that points referring to the same calendar period but based on more distant retrospective reports do not appear, overall, to be located further above range than points based on more recent retrospective periods. This provides further evidence for the veracity of this pattern in sub-Saharan Africa and the ability of the WFS/DHS data to detect it.

A number of epidemiological factors have been proposed in the literature to explain this specific pattern of high _4_ *q*_1_ values in sub-Saharan Africa. Drawing from Senegalese surveillance data, Garenne [9] attributes the unusual level of _4_ *q*_1_ relative to _1_ *q*_0_ to a combination of two main factors: (1) a particular disease environment characterized by high prevalence of malaria, measles, and diarrhea, diseases that appear to generate excess mortality well beyond a child's first birthday; (2) a late age at weaning (centered around 24 mo), combined with elevated mortality around weaning because of the loss of protection from breast milk and inadequate weaning foods. Generalizing to seven surveillance sites in areas of sub-Saharan African countries (in Tanzania, Kenya, Burkina Faso, Mozambique, and Ghana) where malaria is endemic, Abdullah et al. [35] found that the risk of mortality increases with age following an initial decrease during the first few months of life, and they attributed this increase to malaria mortality. Compared to populations where mortality declines monotonically with age, malaria thus appears to shift the distribution of under-five deaths towards older ages within that interval, contributing to relatively high levels of _4_ *q*_1_.

**Figure 12:**
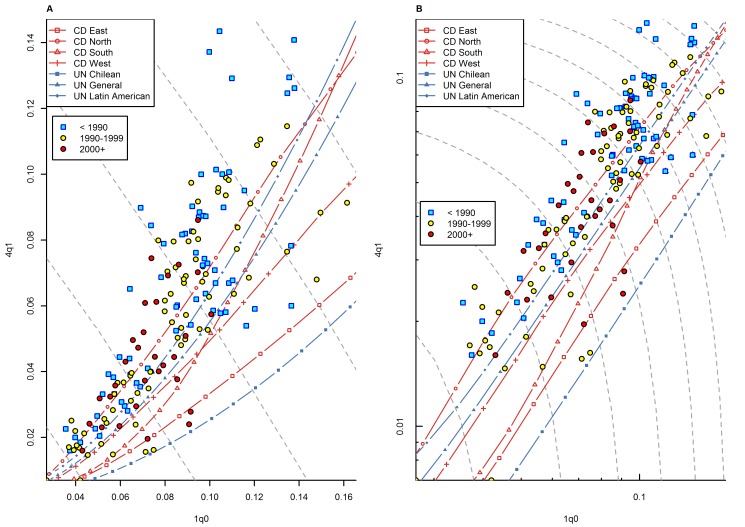
Relationship between _1_ *q*_0_ and _4_ *q*_1_ in WFS/DHS data for countries with high current prevalence of HIV (>5%), by time period (<1990, 1990s, 2000+). (A) Linear scale; (B) logarithmic scale. Countries included: Botswana, Burundi, Cameroon, Central African Republic, Congo, Côte d'Ivoire, Gabon, Kenya, Lesotho, Malawi, Mozambique, Namibia, Rwanda, South Africa, Swaziland, Tanzania, Uganda, Zambia, and Zimbabwe. Gray dashed lines represent isoclines for combinations of _1_ *q*_0_ and _4_ *q*_1_ that produce an identical level of _5_ *q*_0_. CD, Coale and Demeny.

Another explanation that may be invoked for the specific pattern of high _4_ *q*_1_ in certain regions of sub-Saharan Africa is HIV/AIDS. Indeed, various studies have shown that HIV-infected children experience excess mortality during their first 5 y and beyond [36],[37]. However, the age pattern of mortality for HIV-infected babies is complex and depends on whether babies are infected in utero, intranatally, or postnatally [37]. Overall, it is not clear whether a high prevalence of HIV contributes to unusually high levels of _4_ *q*_1_ relative to _1_ *q*_0_. A detailed analysis of this issue is beyond the scope of this paper, but an overview of patterns of infant and child mortality in countries with high HIV prevalence suggests that the emergence of HIV has not substantially modified preexisting patterns of _1_ *q*_0_ versus _4_ *q*_1_. Figure 12 presents the _1_ *q*_0_ versus _4_ *q*_1_ relationship for sub-Saharan African countries that are currently experiencing high prevalence of HIV (above 5%). For most of these countries, HIV was virtually nonexistent prior to the late 1980s/early 1990s. HIV prevalence increased rapidly during the 1990s, and in most cases reached peak values in the late 1990s/early 2000s. If HIV substantially modified the level of _4_ *q*_1_ relative to _1_ *q*_0_, patterns would be clearly different for the pre-1990 versus the post-2000 period. However, for many of these countries, unusually high levels of _4_ *q*_1_ were observed *prior* to the emergence of HIV, and patterns of deviation for the years 2000 and later do not appear to be different from those of earlier time periods. Also, we note that in South Africa, where the prevalence of HIV is very high (18%), the _1_ *q*_0_ versus _4_ *q*_1_ relationship fits rather well within existing model patterns in the DHS data (Figure 10). While HIV may be contributing to high relative values of _4_ *q*_1_, its emergence does not appear to have substantially modified preexisting patterns of _4_ *q*_1_ versus _1_ *q*_0_.

### Understanding Below-Range Values of _4_ *q*_1_

A few countries present unusually low values of _4_ *q*_1_ relative to _1_ *q*_0_, most notably Brazil, Jordan, Turkey, and former Soviet republics of central Asia and the Caucasus. It is unlikely that data quality issues are the main reason for this pattern, because the most commonly discussed types of data errors in DHS surveys (underreporting of infant deaths and age heaping around age 1 y) generate high rather than low relative values of _4_ *q*_1_. It may be hypothesized that this pattern of low relative _4_ *q*_1_ arises in populations that have experienced rapid progress in the area of infectious diseases, but where progress in the area of obstetric practices and neonatal care has lagged.

## Discussion

In this study, we found that, on the whole, empirical values _1_ *q*_0_ and _4_ *q*_1_ fall relatively well within the range provided by Coale and Demeny and UN model life tables, but we also found important exceptions. Sub-Saharan African countries have a tendency to exhibit high values of _4_ *q*_1_ relative to _1_ *q*_0_, a pattern that appears to arise for the most part from true epidemiological causes. While this pattern is well known in the case of western Africa, we observed that it is more widespread than commonly thought. We also found that the emergence of HIV/AIDS, while perhaps contributing to high relative values of _4_ *q*_1_, does not appear to have substantially modified preexisting patterns of under-five mortality. We also identified a small number of countries scattered in different parts of the world that exhibit unusually low values of _4_ *q*_1_ relative to _1_ *q*_0_, a pattern that is not likely to arise merely from data errors. Finally, we illustrated that it is relatively common for populations to experience changes in age patterns of infant and child mortality as they experience a decline in mortality.

Since existing model life tables do not appear to cover the entire range of epidemiological situations and trajectories, they should be used with caution for estimating _1_ *q*_0_ and _4_ *q*_1_ on the basis of _5_ *q*_0_. In particular, we consider that there is little ground for using the same model over a long time period for a given country. In countries like Indonesia where the slope of decline in relative _4_ *q*_1_ has been faster than predicted by model life tables, the use of models may estimate faster declines in _1_ *q*_0_ than experienced in reality. For many sub-Saharan African countries, it is quite clear that the use of existing model life tables yields incorrect infant and child mortality information. Specifically, when using _5_ *q*_0_ as the input, existing models in many cases predict values of _1_ *q*_0_ that are too high and values of _4_ *q*_1_ that are too low.

When observed values of _1_ *q*_0_ and _4_ *q*_1_ systematically deviate from existing model life tables, there is no hard and fast rule for deciding whether the data should be taken at face value or corrected using the model life tables, and, if the latter, which family should be used for the correction. In this global overview, we found that in sub-Saharan Africa, above-range deviations arise primarily from true epidemiological patterns rather than from data errors. This does not mean that this is true in all instances where above-range deviations are reported. Data errors may also contribute to some extent to the patterns observed in sub-Saharan Africa, and may actually exaggerate true epidemiological patterns. Interpretation of above-range deviations should be guided by knowledge of the disease environment. Unusually high values of _4_ *q*_1_ seem to be prevalent in high-mortality populations with little or no control over the surrounding disease environment. The presence of this pattern in populations that don't fit this description should be regarded with suspicion.

Further research is needed to determine the exact contribution of data errors in the observed patterns for DHS/WFS countries. While some procedures for correcting for age heaping have been proposed [19],[21], these procedures should be systematically evaluated, and their impact on age patterns of infant and child mortality should be examined. It may also be worthwhile to examine more detailed age groups in the DHS surveys in order to better understand the sources of deviation, and to distinguish between errors arising from omission of demographic events and errors arising from age misreporting.

This global overview also clearly shows the need to expand existing model life tables so that (1) they may cover the unusually high _4_ *q*_1_ situations observed in many sub-Saharan countries; (2) they may better capture the changes in age patterns experienced by populations as they transition from high to low mortality. While some proposals for modeling sub-Saharan African patterns of infant and child mortality have been made [21], they have not reached the broader scientific community and are not readily integrated into computer programs for mortality estimation such as MortPak [27]. The development of additional age patterns of infant and child mortality that could be routinely used in indirect estimation methods is urgently needed.

This overview also stresses the importance of providing values of _1_ *q*_0_ and _4_ *q*_1_, rather than relying solely on _5_ *q*_0_. First, examining the observed _1_ *q*_0_ versus _4_ *q*_1_ pattern in a population can help detect data errors, including omission errors that also affect the level of _5_ *q*_0_. Second, this overview shows that there is a wide range of _1_ *q*_0_ and _4_ *q*_1_ value combinations that can occur at a given level of _5_ *q*_0_. The level of _4_ *q*_1_ relative to _1_ *q*_0_ for a particular population reflects important epidemiological processes, and thus it is important to convey this information whenever possible.

## Supporting Information

Text S1 **List of countries and years analyzed.**
(DOC)

## Abbreviations

_1_*q*_0_: infant mortality rate
_4_*q*_1_: child mortality rate
_5_*q*_0_: under-five mortality rate
DHS: Demographic and Health Surveys
DSS: Demographic Surveillance Systems
HMD: Human Mortality Database
ISO: International Organization for Standardization
UN: United Nations
WFS: World Fertility Survey
