# Supplementary material for: Child Mortality Estimation: A Global Overview of Infant and Child Mortality Age Patterns in Light of New Empirical Data
Source: PLoS Med. 2012 Aug 28;9(8):e1001299. doi: 10.1371/journal.pmed.1001299 (PMC3429403; doi:10.1371/journal.pmed.1001299)
Supplement: Text S1 — List of countries and years analyzed. (DOC) [file pmed.1001299.s001.doc]

**Text S1: List of countries and years analyzed**

Human Mortality Database

| **Region** | **Country and years of observation** |
| --- | --- |
| **Eastern Europe:** | Belarus: 1960-2010 |
|  | Bulgaria: 1947-2010 |
|  | Czech Republic: 1950-2010 |
|  | German Democratic Republic: 1956-2009 |
|  | Hungary: 1950-2010 |
|  | Poland: 1958-2010 |
|  | Russian Federation: 1960-2010 |
|  | Slovakia: 1950-2010 |
|  | Ukraine: 1960-2010 |
| **Northern Europe:** | Denmark: 1835-2010 |
|  | Estonia: 1960-2010 |
|  | Finland: 1878-2010 |
|  | Iceland: 1838-2010 |
|  | Ireland: 1950-2010 |
|  | Latvia: 1960-2010 |
|  | Lithuania: 1960-2010 |
|  | Norway: 1846-2010 |
|  | Sweden: 1751-2010 |
|  | United Kingdom - England and Wales: 1841-2010 |
|  | United Kingdom - Northern Ireland: 1922-2010 |
|  | United Kingdom - Scotland: 1855-2010 |
| **Southern Europe :** | Italy: 1872-2009 |
|  | Portugal: 1940-2010 |
|  | Slovenia: 1983-2010 |
|  | Spain: 1908-2010 |
| **Western Europe :** | Austria: 1947-2010 |
|  | Belgium: 1841-2010 |
|  | France: 1816-2010 |
|  | Germany (Federal Republic of): 1956-2009 |
|  | Luxembourg: 1960-2010 |
|  | Netherlands: 1850-2010 |
|  | Switzerland: 1876-2010 |
| **Other OECD countries:** | Australia: 1921-2008 |
|  | Canada: 1921-2008 |
|  | Chile: 1992-2005 |
|  | Israel: 1983-2010 |
|  | Japan: 1947-2010 |
|  | New Zealand: 1901-2009 |
|  | United States of America: 1933-2008 |

World Fertility Survey (WFS) and Demographic Health Surveys (DHS)

| **Region** | **Country (ISO3 code)** | **Survey**^[a]^ | **Retrospective period** |
| --- | --- | --- | --- |
|
| **Asia** | Bangladesh (BGD) | 1975-1976 WFS | 1960-1975 |
|  |  | 1993-1994 DHS | 1978-1993 |
|  |  | 1996-1997 DHS | 1981-1996 |
|  |  | 1999-2000 DHS | 1984-1999 |
|  |  | 2004 DHS | 1989-2004 |
|  |  | 2007 DHS | 1992-2007 |
|  | Cambodia (KHM) | 2000 DHS | 1985-2000 |
|  |  | 2005 DHS | 1990-2005 |
|  |  | 2010 DHS | 1995-2010 |
|  | Fiji (FJI) | 1974 WFS | 1959-1974 |
|  | India (IND) | 1992-1993 DHS | 1977-1992 |
|  |  | 1998-1999 DHS | 1984-1999 |
|  |  | 2005-2006 DHS | 1991-2006 |
|  | Indonesia (IDN) | 1976 WFS | 1961-1976 |
|  |  | 1987 DHS | 1972-1987 |
|  |  | 1991 DHS | 1976-1991 |
|  |  | 1994 DHS | 1979-1994 |
|  |  | 1997 DHS | 1982-1997 |
|  |  | 2002-2003 DHS | 1987-2002 |
|  |  | 2007 DHS | 1992-2007 |
|  | Maldives (MDV) | 2009 DHS | 1994-2009 |
|  | Nepal (NPL) | 1996 DHS | 1980-1995 |
|  |  | 2001 DHS | 1979-1994 |
|  |  | 2006 DHS | 1990-2005 |
|  | Pakistan (PAK) | 1990-1991 DHS | 1975-1990 |
|  |  | 2006-2007 DHS | 1991-2006 |
|  | Philippines (PHL) | 1978 WFS | 1963-1978 |
|  |  | 1993 DHS | 1978-1993 |
|  |  | 1998 DHS | 1983-1998 |
|  |  | 2003 DHS | 1988-2003 |
|  |  | 2008 DHS | 1993-2008 |
|  | Republic of Korea (KOR) | 1974 WFS | 1959-1974 |
|  | Samoa (WSM) | 2009 DHS | 1993-2008 |
|  | Sri Lanka (LKA) | 1987 DHS | 1971-1986 |
|  | Thailand (THA) | 1987 DHS | 1972-1987 |
|  | Timor-Leste (TLS) | 2009 DHS | 1994-2009 |
|  | Viet Nam (VNM) | 1997 DHS | 1982-1997 |
|  |  | 2002 DHS | 1987-2002 |
| **Former Soviet Union** | Armenia (ARM) | 2000 DHS | 1985-2000 |
|  |  | 2005 DHS | 1990-2005 |
|  | Azerbaijan (AZE) | 2006 DHS | 1991-2006 |
|  | Kazakhstan (KAZ) | 1995 DHS | 1980-1995 |
|  |  | 1999 DHS | 1984-1999 |
|  | Kyrgyzstan (KGZ) | 1997 DHS | 1982-1997 |
|  | Republic of Moldova (MDA) | 2005 DHS | 1990-2005 |
|  | Turkmenistan (TKM) | 2000 DHS | 1984-1999 |
|  | Ukraine (UKR) | 2007 DHS | 1992-2007 |
|  | Uzbekistan (UZB) | 1996 DHS | 1981-1996 |
| **Latin America  and the Caribbean** | Bolivia (BOL) | 1989 DHS | 1974-1989 |
|  |  | 1994 DHS | 1979-1994 |
|  |  | 1998 DHS | 1983-1998 |
|  |  | 2003 DHS | 1988-2003 |
|  |  | 2008 DHS | 1993-2008 |
|  | Brazil (BRA) | 1991 DHS | 1976-1991 |
|  |  | 1996 DHS | 1981-1996 |
|  | Colombia (COL) | 1990 DHS | 1975-1990 |
|  |  | 1995 DHS | 1980-1995 |
|  |  | 2000 DHS | 1985-2000 |
|  |  | 2005 DHS | 1990-2005 |
|  |  | 2010 DHS | 1995-2010 |
|  | Dominican Republic (DOM) | 1991 DHS | 1976-1991 |
|  |  | 1996 DHS | 1981-1996 |
|  |  | 1999 DHS | 1984-1999 |
|  |  | 2002 DHS | 1987-2002 |
|  |  | 2007 DHS | 1992-2007 |
|  |  | 2007a DHS | 1992-2007 |
|  | Ecuador (ECU) | 1979-1980 WFS | 1963-1978 |
|  |  | 1987 DHS | 1971-1986 |
|  | El Salvador (SLV) | 1985 DHS | 1970-1985 |
|  | Guatemala (GTM) | 1987 DHS | 1972-1987 |
|  |  | 1995 DHS | 1980-1995 |
|  |  | 1998-1999 DHS | 1983-1998 |
|  | Guyana (GUY) | 1975 WFS | 1960-1975 |
|  |  | 2005 AIS | 1990-2005 |
|  |  | 2009 DHS | 1994-2009 |
|  | Haiti (HTI) | 1977 WFS | 1962-1977 |
|  |  | 1994-1995 DHS | 1979-1994 |
|  |  | 2000 DHS | 1985-2000 |
|  |  | 2005-2006 DHS | 1990-2005 |
|  | Honduras (HND) | 2005-2006 DHS | 1991-2006 |
|  | Jamaica (JAM) | 1976 WFS | 1960-1975 |
|  | Mexico (MEX) | 1976-1977 WFS | 1966-1981 |
|  |  | 1987 DHS | 1972-1987 |
|  | Nicaragua (NIC) | 1998 DHS | 1982-1997 |
|  |  | 2001 DHS | 1986-2001 |
|  | Paraguay (PRY) | 1990 DHS | 1975-1990 |
|  | Peru (PER) | 1977-1978 WFS | 1963-1978 |
|  |  | 1991-1992 DHS | 1976-1991 |
|  |  | 1996 DHS | 1981-1996 |
|  |  | 2000 DHS | 1985-2000 |
|  | Trinidad and Tobago (TTO) | 1977 WFS | 1962-1977 |
|  |  | 1987 DHS | 1972-1987 |
| **Middle East  and North Africa** | Egypt (EGY) | 1980 1980 | 1964-1979 |
|  |  | 1988 DHS | 1973-1988 |
|  |  | 1991 PAPCHILD | 1976-1986 |
|  |  | 1992 DHS | 1977-1992 |
|  |  | 1995 DHS | 1980-1995 |
|  |  | 1997 DHS | 1982-1997 |
|  |  | 1998 Interim DHS | 1981-1996 |
|  |  | 2000 DHS | 1985-2000 |
|  |  | 2003 DHS | 1988-2003 |
|  |  | 2005 DHS | 1990-2005 |
|  |  | 2008 DHS | 1993-2008 |
|  | Jordan (JOR) | 1990 DHS | 1975-1990 |
|  |  | 1997 DHS | 1982-1997 |
|  |  | 2002 DHS | 1987-2002 |
|  |  | 2007 DHS | 1992-2007 |
|  |  | 2009 DHS | 1994-2009 |
|  | Morocco (MAR) | 1980 WFS | 1965-1980 |
|  |  | 1987 DHS | 1972-1987 |
|  |  | 1992 DHS | 1977-1992 |
|  |  | 1995 DHS | 1979-1994 |
|  |  | 2003-2004 DHS | 1988-2003 |
|  | Sudan (SDN) | 1978-1979 WFS | 1963-1978 |
|  |  | 1989-1990 DHS | 1974-1989 |
|  | Syrian Arab Republic (SYR) | 1978 WFS | 1963-1978 |
|  | Tunisia (TUN) | 1978 WFS | 1963-1978 |
|  |  | 1988 DHS | 1973-1988 |
|  | Turkey (TUR) | 1978 WFS | 1963-1978 |
|  |  | 1993 DHS | 1978-1993 |
|  |  | 1998 DHS | 1983-1998 |
|  |  | 2003 DHS | 1988-2003 |
|  | Yemen (YEM) | 1979 WFS | 1964-1979 |
|  |  | 1991-1992 DHS | 1976-1991 |
|  |  | 1997 DHS | 1981-1996 |
| **Sub-Saharan Africa** | Angola (AGO) | 2006-2007 MIS | 2001-2006 |
|  | Benin (BEN) | 1996 DHS | 1981-1996 |
|  |  | 2001 DHS | 1986-2001 |
|  |  | 2006 DHS | 1991-2006 |
|  | Botswana (BWA) | 1998 DHS | 1972-1987 |
|  | Burkina Faso (BFA) | 1993 DHS | 1977-1992 |
|  |  | 1998-1999 DHS | 1983-1998 |
|  |  | 2003 DHS | 1988-2003 |
|  | Burundi (BDI) | 1987 DHS | 1972-1987 |
|  | Cameroon (CMR) | 1978 WFS | 1963-1978 |
|  |  | 1991 DHS | 1976-1991 |
|  |  | 1998 DHS | 1983-1998 |
|  |  | 2004 DHS | 1989-2004 |
|  | Central African Republic (CAF) | 1994-1995 DHS | 1979-1994 |
|  | Chad (TCD) | 1996-1997 DHS | 1982-1997 |
|  |  | 2004 DHS | 1989-2004 |
|  | Comoros (COM) | 1996 DHS | 1981-1996 |
|  | Congo (COG) | 2005 DHS | 1990-2005 |
|  | Côte d'Ivoire (CIV) | 1994 DHS | 1979-1994 |
|  |  | 1998-1999 DHS | 1983-1998 |
|  |  | 2005 AIS | 1990-2005 |
|  | Dem. Rep. of the Congo (COD) | 2007 DHS | 1992-2007 |
|  | Eritrea (ERI) | 1995 DHS | 1979-1994 |
|  | Ethiopia (ETH) | 2000 DHS | 1977-1992 |
|  |  | 2005 DHS | 1982-1997 |
|  | Gabon (GAB) | 2000 DHS | 1985-2000 |
|  | Ghana (GHA) | 1979-1980 WFS | 1964-1979 |
|  |  | 1988 DHS | 1973-1988 |
|  |  | 1993 DHS | 1978-1993 |
|  |  | 1998 DHS | 1983-1998 |
|  |  | 2003 DHS | 1988-2003 |
|  |  | 2008 DHS | 1993-2008 |
|  | Guinea (GIN) | 1992 DHS | 1976-1991 |
|  |  | 1999 DHS | 1984-1999 |
|  |  | 2005 DHS | 1990-2005 |
|  | Kenya (KEN) | 1977-1978 WFS | 1962-1977 |
|  |  | 1989 DHS | 1974-1989 |
|  |  | 1993 DHS | 1978-1993 |
|  |  | 1998 DHS | 1983-1998 |
|  |  | 2003 DHS | 1988-2003 |
|  |  | 2008-2009 DHS | 1993-2008 |
|  | Lesotho (LSO) | 1977 WFS | 1962-1977 |
|  |  | 2004 DHS | 1989-2004 |
|  |  | 2009 DHS | 1994-2009 |
|  | Liberia (LBR) | 2007 DHS | 1991-2006 |
|  |  | 2009 MIS | 1993-2008 |
|  | Madagascar (MDG) | 1992 DHS | 1977-1992 |
|  |  | 1997 DHS | 1982-1997 |
|  |  | 2003-2004 DHS | 1988-2003 |
|  |  | 2008-2009 DHS | 1993-2008 |
|  | Malawi (MWI) | 1992 DHS | 1977-1992 |
|  |  | 2000 DHS | 1985-2000 |
|  |  | 2004 DHS | 1989-2004 |
|  |  | 2010 DHS | 1995-2010 |
|  | Mali (MLI) | 1987 DHS | 1972-1987 |
|  |  | 1995-1996 DHS | 1980-1995 |
|  |  | 2001 DHS | 1986-2001 |
|  |  | 2006 DHS | 1991-2006 |
|  | Mauritania (MRT) | 1981 WFS | 1966-1981 |
|  |  | 2003 EMIP | 1988-2003 |
|  | Mozambique (MOZ) | 1997 DHS | 1982-1997 |
|  |  | 2003 DHS | 1988-2003 |
|  | Namibia (NAM) | 1992 DHS | 1977-1992 |
|  |  | 2000 DHS | 1985-2000 |
|  |  | 2006-2007 DHS | 1991-2006 |
|  | Niger (NER) | 1992 DHS | 1977-1992 |
|  |  | 1998 DHS | 1983-1998 |
|  |  | 2006 DHS | 1991-2006 |
|  | Rwanda (RWA) | 1992 DHS | 1977-1992 |
|  |  | 2000 DHS | 1985-2000 |
|  |  | 2005 DHS | 1990-2005 |
|  |  | 2007-2008 DHS | 1993-2008 |
|  | Senegal (SEN) | 1978 WFS | 1963-1978 |
|  |  | 1992-1993 DHS | 1977-1992 |
|  |  | 1997 DHS | 1982-1997 |
|  |  | 2005 DHS | 1990-2005 |
|  |  | 2008-2009 MIS | 1993-2008 |
|  | Sierra Leone (SLE) | 2008 DHS | 1993-2008 |
|  | South Africa (ZAF) | 1998 DHS | 1983-1998 |
|  |  | 2003 DHS | 1987-2002 |
|  | Swaziland (SWZ) | 2006-2007 DHS | 1991-2006 |
|  | São Tomé and Príncipe (STP) | 2008-2009 DHS | 1993-2008 |
|  | Togo (TGO) | 1988 DHS | 1973-1988 |
|  |  | 1998 DHS | 1983-1998 |
|  | Uganda (UGA) | 1988-1989 DHS | 1973-1988 |
|  |  | 1995 DHS | 1980-1995 |
|  |  | 2000-2001 DHS | 1985-2000 |
|  |  | 2006 DHS | 1991-2006 |
|  |  | 2009 MIS | 1994-2009 |
|  | UR of Tanzania (TZA) | 1991-1992 DHS | 1976-1991 |
|  |  | 1995 SUMVE | 1979-1994 |
|  |  | 1996 DHS | 1981-1996 |
|  |  | 1999 DHS | 1984-1999 |
|  |  | 2004-2005 DHS | 1989-2004 |
|  |  | 2007-2008 AIS | 1992-2007 |
|  |  | 2010 DHS | 1995-2010 |
|  | Zambia (ZMB) | 1992 DHS | 1977-1992 |
|  |  | 1996 DHS | 1981-1996 |
|  |  | 2001-2002 DHS | 1986-2001 |
|  |  | 2007 DHS | 1992-2007 |
|  | Zimbabwe (ZWE) | 1988 DHS | 1973-1988 |
|  |  | 1994 DHS | 1979-1994 |
|  |  | 1999 DHS | 1984-1999 |
|  |  | 2005-2006 DHS | 1990-2005 |

[a] Note: AIS (AIDS Indicator Surveys), EMIP (Enquête sur la mortalité infantile et le paludisme), MIS (Malaria Indicators Surveys), SUMVE (In-depth study on estimating adult and childhood mortality in settings of high adult mortality)

Demographic Surveillance Sites: Altogether this analysis reviews 127 mortality rates for different period through 1930-2006 across 28 sites in sub-Saharan Africa (18 sites in Western Africa, 9 sites in Eastern Africa, and 1 in Southern Africa). The detailed list is as follow:

| **Region** | **Country** | **Sites** |
| --- | --- | --- |
| **West Africa** |  |  |
|  | Burkina Faso: | - 1993-2001 Nouna - 2002-2004 Ouagadougou - 1994-1998 Oubritenga |
|  | Gambia: | - 1994-1999 Farafenni - 1943-1997 Keneba - 1989-1993 Upper River Division |
|  | Ghana: | - 1974-1977 Cape Coast - 1971-1972 Danfa - 1993-1999 Navrongo |
|  | Guinea-Bissau: | - 1990-1999 Bandim |
|  | Nigeria: | - 1974-1977 Malumfashi |
|  | Senegal: | - 1970-1999 Bandafassi - 1943-1965 Fakao - 1930-1999 Mlomp - 1963-1991 N'gayokhème - 1963-1998 Niakhar - 1963-1965 Paos-Koto - 1975-1979 Peul Bande |
| **Eastern Africa** | Ethiopia: | - 1987-1999 Butajira |
|  | Kenya: | - 1975-1978 Machakos |
|  | Mozambique: | - 1995-1999 Manhica |
|  | Tanzania: | - 1984-1985 Kikwawila - 1992-1999 Hai and Morogoro - 1995-1999 Ifakara and Rufiji |
|  | Zambia: | - 1950-1999 Gwembe |
| **Southern Africa** | South Africa: | - 1992-1999 Agincourt in |

**References:**
